# Differential Diagnosis of Histiocytic Necrotizing Lymphadenitis and Malignant Lymphoma with Simple Clinical Findings

**DOI:** 10.3390/children9020290

**Published:** 2022-02-20

**Authors:** Taichi Omachi, Naho Atsumi, Takashi Yamazoe, Sohsaku Yamanouchi, Ryosuke Matsuno, Tomoki Kitawaki, Kazunari Kaneko

**Affiliations:** 1 Department of Pediatrics, Kansai Medical University, 2-5-1 Shin-machi, Hirakata, Osaka 573-1010, Japan; omachit@hirakata.kmu.ac.jp (T.O.); yamataka0919@gmail.com (T.Y.); yamanous@hirakata.kmu.ac.jp (S.Y.); matsunor@hirakata.kmu.ac.jp (R.M.); 2 Department of Pathology, Kansai Medical University, 2-5-1 Shin-machi, Hirakata, Osaka 573-1010, Japan; naatsumi@hirakata.kmu.ac.jp; 3 Department of Mathematics, Kansai Medical University, 2-5-1 Shin-machi, Hirakata, Osaka 573-1010, Japan; kitawaki@hirakata.kmu.ac.jp

**Keywords:** lymphadenitis, body temperature, maximum lymph node size, β_2_-microglobulin

## Abstract

It is desirable that noninvasive differential diagnosis takes place without lymph node biopsy for histiocytic necrotizing lymphadenitis (HNL) or malignant lymphoma (ML). In this study, we propose a novel scoring model for the differential diagnosis of these diseases using clinical information and clinical findings. We retrospectively analyzed the data from 15 HNL and 13 ML pediatric patients. First, a univariate analysis identified 14 clinical factors with significant differences. Second, a subsequent analysis using receiver operating characteristic (ROC) curve analysis identified three factors among them with area under the ROC curve values of >0.95: body temperature (°C), maximum lymph node size (cm), and serum β_2_-microglobulin level (mg/L). Finally, the cut-off values of each of these three factors were determined and examined for the 28 cases. All 15 HNL cases were within 2–3 of the cut-off values among the three factors, no ML case was within two or more cut-off values. Thus, the diagnostic sensitivity and specificity of this novel scoring system were both 100%, indicating that clinical scoring with body temperature, maximum lymph node size, and β_2_-microglobulin are useful for distinguishing between HNL and ML.

## 1. Introduction

Histiocytic necrotizing lymphadenitis (HNL), also known as Kikuchi–Fujimoto disease, was first reported by Kikuchi and Fujimoto in 1972 as “lymphadenitis showing focal reticulum cell hyperplasia with nuclear debris and phagocytosis” [1]. This disease is frequently observed in Asia [1]. Because HNL is characterized by fever and lymphadenitis [1,2], it is important to distinguish it from childhood diseases that also show fever and lymphadenopathy as common findings, including viral infections such as cytomegalovirus (CMV), Epstein–Barr virus (EBV), and herpes simplex virus, as well as Kawasaki disease, autoimmune diseases such as systemic erythematosus, and malignant lymphoma (ML) [2,3]. Among these, viral infections can be differentially diagnosed using serum antibody titers. Kawasaki disease has characteristic symptoms in addition to fever and lymphadenopathy, all of which are used as diagnostic criteria [4]. Autoimmune diseases can also be diagnosed not only by symptoms caused by autoinflammatory reactions such as a rash but also through the detection of autoantibodies by serological tests. Conversely, it is difficult to differentially diagnose ML and HNL because they share common clinical symptoms and routine laboratory findings [5]. Because HNL is a benign and self-limiting disease, treatment is symptomatic, and antipyretic analgesics, nonsteroidal anti-inflammatory drugs, and (in rare cases) corticosteroids are used [6]. In contrast, ML patients require chemotherapy, which is administered following a histological diagnosis that necessitates an invasive lymph node (LN) biopsy. Thus, a noninvasive differential diagnosis without LN biopsy is undoubtedly beneficial for pediatric patients with HNL.

This study was conducted to propose a novel scoring model for the differential diagnosis of HNL from ML using clinical information and clinical findings.

## 2. Materials and Methods

This study enrolled patients under 18 years old diagnosed with HNL (*n* = 15) or ML (*n* = 13) who were admitted to Kansai Medical University Hospital between 1 January 2006 and 31 August 2021, and their clinical information and findings were retrospectively assessed. Patients with HNL or ML were diagnosed according to the histological findings of biopsied LNs. The pathological breakdown of ML was T cell lymphoblastic lymphoma (*n* = 6), B cell lymphoblastic lymphoma (*n* = 2), Hodgkin lymphoma (*n* = 2), Burkitt lymphoma (*n* = 1), diffuse large B cell lymphoma (*n* = 1), and anaplastic large cell lymphoma (*n* = 1), and the clinical stages were Stage 1 (*n* = 2), Stage 2 (*n* = 2), Stage 3 (*n* = 7), and Stage 4 (*n* = 2). This study was conducted with the approval of the ethics committee of Kansai Medical University Hospital, and all data were anonymized.

**Figure 1:**
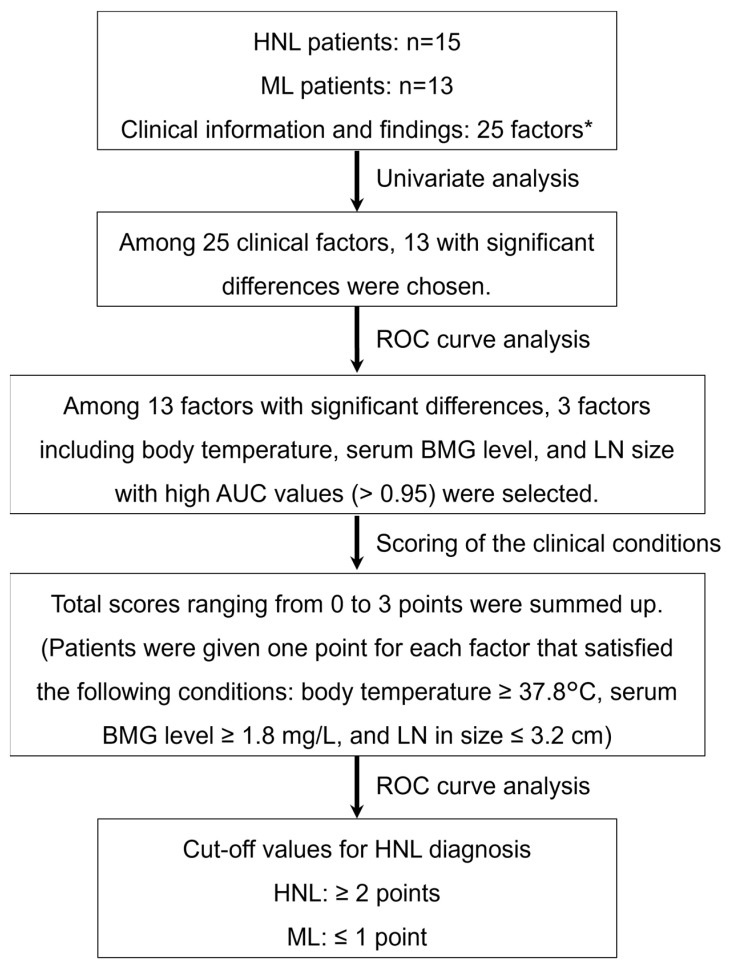
Flowchart of the establishment of a new scoring method for the differential diagnosis of HNL and ML. * The 25 factors consisting of clinical information and findings were as follows: clinical findings included body temperature, duration of fever, lymph node (LN) size, LN tenderness, and skin rash; blood tests included white blood cell count (WBC), neutrophil count, monocyte count, lymphocyte count, atypical lymphocyte count, red blood cell (RBC) count, hemoglobin (Hb), hematocrit (HCT), mean corpuscular volume (MCV), red blood cell distribution width (RDW), blood platelet count (Plt), serum levels of aspartate transaminase (AST), alanine aminotransferase (ALT), triacylglycerol (TG), lactic dehydrogenase (LDH), sodium (Na), C-reactive protein (CRP), β_2_-microglobulin (BMG), ferritin, and soluble IL-2 receptor (sIL-2R). HNL, histiocytic necrotizing lymphadenitis; ML, malignant lymphoma; ROC curve analysis, receiver operating characteristic curve analysis; AUC, area under the ROC curve.

A flowchart of the proposed new scoring method is shown in Figure 1. Clinical findings (body temperature (°C), duration of fever (days), LN size (cm), presence or absence of LN tenderness, and skin rash) were extracted from the patients’ medical records. Maximum LN diameter was precisely measured according to computed tomography (CT) or magnetic resonance imaging (MRI). Of the blood test findings, white blood cell count (WBC), neutrophil count, monocyte count, lymphocyte count, atypical lymphocyte count, red blood cell (RBC) count, hemoglobin (Hb), hematocrit (HCT), mean corpuscular volume (MCV), red blood cell distribution width (RDW), blood platelet count (Plt), serum levels of aspartate transaminase (AST), alanine aminotransferase (ALT), triacylglycerol (TG), lactic dehydrogenase (LDH), sodium (Na), C-reactive protein (CRP), β_2_-microglobulin (BMG), ferritin, and soluble IL-2 receptor (sIL-2R) were compared.

First, univariate analysis was performed, with *p* < 0.05 considered statistically significant according to Fisher’s exact test, for the presence of LN tenderness and skin rash and the Mann–Whitney U test for 23 continuous variables, as shown in Figure 1. Then, receiver operating characteristic (ROC) curve analysis was performed for the factors that showed significant differences between HNL and ML. Among them, factors with area under the ROC curve (AUC) values higher than 0.95 were selected and determined to be useful for differential diagnosis. The cut-off value for each of the selected factors was calculated using ROC analysis. Scoring was then performed according to these cut-off values, and each factor was assigned a score of either 0 or 1 point. The discriminatory power of this scoring model was evaluated and verified.

Statistical analysis was performed using R software (version 4.1.0) (R Foundation for Statistical Computing, Vienna, Austria) [7] and R Studio Desktop software (version 1.4.1717) (R Studio, Boston, MA, USA) [8].

## 3. Results

### 3.1. Patients

The median ages of patients with HNL and ML were 10.83 years (range, 5.4–14.8 years) and 10.33 years (range, 0.7–15 years) (*p* = 0.719), respectively, and no difference between the sexes was observed (HNL: men *n* = 9, women *n* = 6; ML: men *n* = 10, women *n* = 3; *p* = 0.826). Nothing notable was observed regarding perinatal history, medical history, or family history. Serological test results for antinuclear antibody, CMV, EBV, and herpes simplex virus were all negative.

### 3.2. Establishment and Evaluation of the Scoring Model

A total of 25 factors were extracted from the patients’ medical records to determine factors for the scoring model (Figure 1 and Table 1 and Table 2). Univariate analysis revealed that 14 of these 25 factors showed significant differences between HNL and ML patients. As shown in Table 1, patients with HNL had a significantly higher body temperature than patients with ML (39.0 °C vs. 36.9 °C; *p* < 0.001) and a longer duration of fever (14.0 days vs. 0.0 days; *p* < 0.001), and the maximum LN diameter of HNL patients was smaller than that of ML patients (1.9 cm vs. 4.0 cm; *p* < 0.001). The proportion of patients with HNL who complained of LN tenderness was greater than that of patients with ML (100.0% vs. 15.4%; *p* < 0.001).

As shown by the laboratory findings presented in Table 2, serum AST, LDH, BMG, and ferritin in patients with HNL were significantly higher than in patients with ML (43 U/L vs. 22 U/L, *p* < 0.01; 449 U/L vs. 260 U/L, *p* = 0.01116; 2.5 mg/L vs. 1.6 mg/L, *p* < 0.001; and 282 ng/mL vs. 35 ng/mL, *p* < 0.001, respectively). In addition, WBC count, neutrophil count, lymphocyte count, RBC count, Plt count, and serum Na were significantly lower in patients with HNL compared with patients with ML (2600/μL vs. 7400/μL, *p* < 0.001; 1711/μL vs. 4030/μL; p<0.005; 1189/μL vs. 3040/μL, *p* < 0.01; 4.64 M/μL vs. 5.02 M/μL, *p* = 0.0295; 1710 × 109/L vs. 2840 × 109/L, *p* < 0.001; and 138 mEq/L vs. 140 mEq/L, *p* < 0.01, respectively).

**Figure 2:**
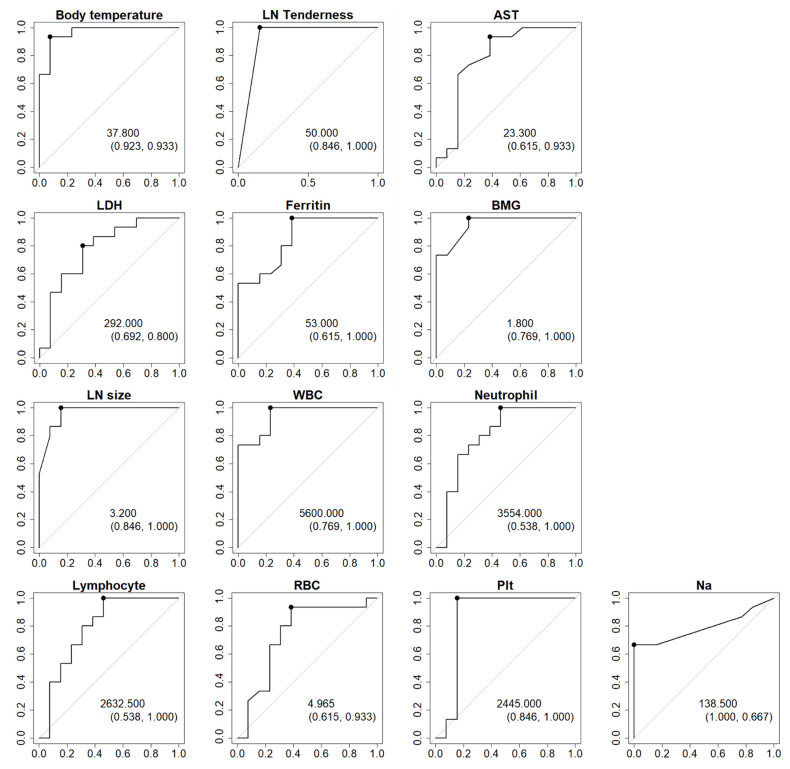
ROC curve analysis of 13 candidate factors with significant differences between HNL and ML. Cut-off values with specificity and sensitivity in parentheses are displayed in each panel. Thresholds are displayed as black dots on each ROC curve. X- and Y-axes of all panels denote 1-specificity and sensitivity, respectively. LN, lymph node; AST, aspartate transaminase; LDH, lactic dehydrogenase; BMG, β_2_-microglobulin; WBC, white blood cell count; RBC, red blood cell count; Plt, blood platelet count.

Next, ROC curve analyses of the 13 factors that demonstrated significant differences between HNL patients and ML patients were performed (Figure 1). Although the number of factors with a significant difference totaled 14, the fever duration was excluded from further analyses because it was not appropriate to wait for defervescence in order to make an early differential diagnosis. ROC curves revealed three high-precision factors for which the AUC value exceeded 0.95, and the cut-off value for each was then calculated (Figure 1 and Figure 2, Table 3). These three factors were body temperature, serum BMG level, and LN size, and the cut-off values to distinguish HNL from ML were ≥37.8 °C, ≥1.8 mg/L, and ≤3.2 cm, respectively. In the present 28 cases (15 HNL and 13 ML), one point was given for each factor that satisfied the conditions described above, resulting in total scores of 0–3 points being assigned to each case. As a result, via this new scoring model, HNL and ML could be distinguished with 100% sensitivity, 100% specificity, 100% positive predictive value, and 100% negative predictive value (Table 4).

We confirmed that this scoring model using the three identified factors (total score) achieved higher specificity and sensitivity compared with the scores obtained with a single one of the three factors, i.e., body temperature, BMG, or LN size, based on the AUC (total score, 1.00; body temperature, 0.9282; BMG, 0.8846; and LN size, 0.9231).

**Figure 3:**
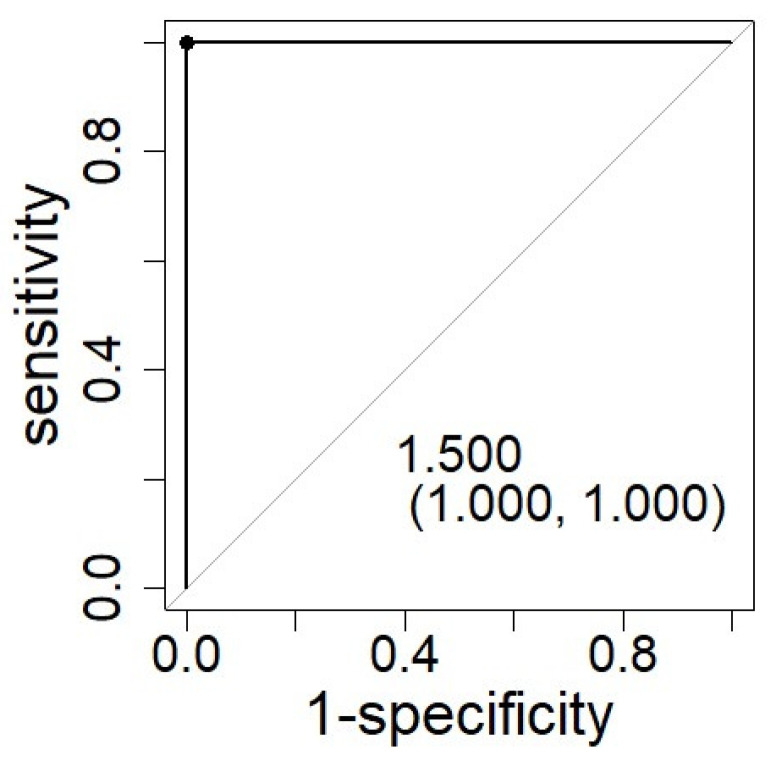
ROC curve analysis of the total scores calculated for 15 HNL cases and 13 ML cases. The cut-off value of the total score is displayed with specificity and sensitivity in parenthesis. Using ROC curve analysis, the cut-off value to distinguish between HNL and ML was shown to be 1.5 points, and this cut-off value yielded an AUC of 1.0 with 100% specificity and 100% sensitivity.

**Figure 4:**
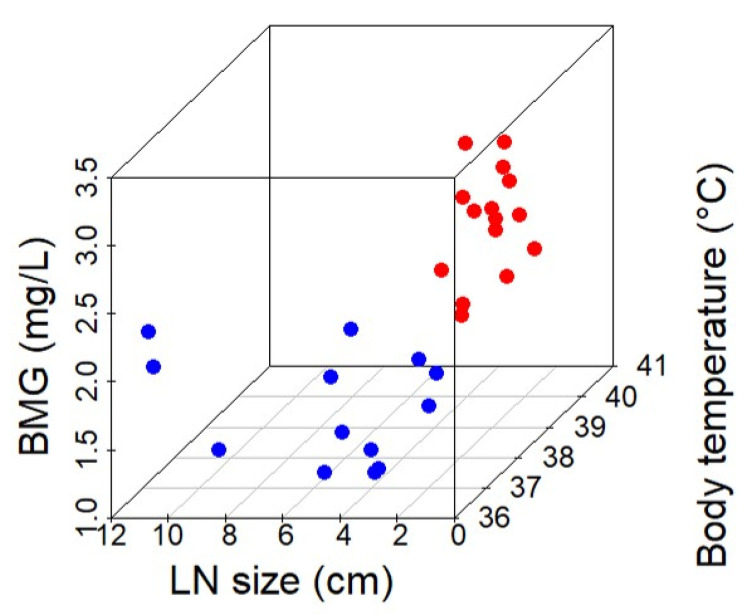
Three-dimensional scatter diagram of three factors in distinguishing between HNL and ML. The X-axis denotes the lymph node size, and Y- and Z-axes indicate body temperature and BMG, respectively. Blue dots and red dots indicate ML and HNL, respectively. HNL, histiocytic necrotizing lymphadenitis; ML, malignant lymphoma; LN, lymph node; BMG, β_2_-microglobulin.

Through ROC curve analysis of this scoring result, the cut-off value to distinguish between HNL and ML was 1.5 points, and this cut-off value yielded an AUC of 1.0 (Figure 3). In other words, if two or more points are given to a patient with a fever and lymphadenopathy in this scoring model, the diagnosis is HNL, and if it is not greater than one point, the diagnosis is ML. The scatterplot of these three factors confirms that HNL cases and ML cases could be clearly distinguished (Figure 4).

## 4. Discussion

In this study, we established a new scoring model to distinguish between HNL and ML in children by considering 25 common clinical factors extracted from their medical records. Univariate and ROC curve analyses identified three factors (body temperature in °C, serum BMG level in mg/L, and LN size in cm) that were considered useful for distinguishing between HNL and ML. Scoring with these three factors achieved 100% sensitivity and 100% specificity, making this model an excellent method for distinguishing between HNL and ML. Compared with previous reports on differential methods that use a single factor [9,10,11], this model comprises items selected from diverse standpoints and can therefore more accurately reflect the true conditions of HNL.

This scoring model is useful for the differential diagnosis of HNL and ML, especially in children, because both HNL and ML are commonly observed in children aged 10–20, whereas it is easy to distinguish the actual clinical site in adults because the age of onset of HNL and ML differ (less than 40 years old and 50 years and older, respectively) [12,13].

We discuss the significance of the three factors (body temperature, serum BMG level, and LN size) considered useful for distinguishing between HNL and ML.

First, regarding fever, it has been reported that fever develops in 70–80% of pediatric patients with HNL [14], whereas it is only observed in 30–50% of adult patients with HNL [6]. Meanwhile, fever is less commonly observed in ML in both adults and children [15]: only 7–18% of patients with ML are found to have a fever [16]. Therefore, the presence of fever as a scoring factor to discriminate HNL from ML may be more valid in children than in adults. To determine whether fever is applicable in adults as well as children, further investigations are required. Regarding the higher body temperature of HNL patients, a previous report using a microarray analysis of peripheral blood mononuclear cells to distinguish between HNL and ML showed that interferon-induced genes were characteristically expressed in HNL [10]. Considering that interferon causes fever, the higher body temperature observed in HNL patients in this study could have resulted from the upregulated expression of interferon-induced genes [10].

In addition, patients with HNL were reported to have a fever for longer than those with ML. According to Lee et al., the median duration of fever in 12 children with HNL was 19.5 days (range, 9–75 days) [17]. In our study, the duration of fever in patients with HNL was significantly longer than that for ML. However, because it was not appropriate to wait for defervescence to make an early differential diagnosis, we considered this difference in fever duration not to be suitable in the scoring model. Consequently, although a significant difference was also observed in the fever duration between HNL and ML patients, this factor was not applied in the scoring model.

Second, in terms of the significance of the serum level of BMG for differential diagnosis, we showed that BMG was increased in all cases of HNL (15/15). Ours is the first study to report higher BMG values in patients with HNL (*n* = 15) compared with ML (*n* = 13) (AUC, 0.9538; specificity, 0.769; sensitivity, 1.000). BMG is secreted from B cells or leukemia cells, resulting in an increase in its serum concentration in cases with viral infection or hematological malignancy [18,19]. Although the prospect of studying a larger number of cases in the future is desirable, we consider the present study to have included enough patients to indicate the potential of serum BMG level (≥1.8 mg/L) as a specific marker for HNL, taking into account the high statistical power (100%) when BMG was compared in 15 HNL and 13 ML cases in the present study.

Third, our study suggests that the LN size is an important factor in distinguishing between benign HNL and aggressive ML. Although ultrasound examination is a noninvasive and effective means to confirm the LN size and to gain valuable characteristics for differential diagnosis (shape, rims, matting, and echotexture of LNs) [9], an objective evaluation is difficult, and a high level of skill is needed for accurate measurement. To overcome this technical disadvantage, the current study used images obtained by CT or MRI to precisely measure the LN size. As a result, these imaging modalities demonstrated a larger LN size in patients with ML than in patients with HNL (median size 1.90 cm in HNL and 4.00 cm in ML; Table 1). Our result is in good agreement with previous reports: the average size of LNs in 96 patients with HNL measured by CT was 1.62 cm [20], while an LN greater than 3.4 cm in size was correlated with a definitive diagnosis of ML [21]. The larger LN size in patients with ML could be caused by the proliferation of leukemia cells contributing to the prominent enlargement of LNs [22], and, in contrast, the immune reaction in HNL patients may have a limited impact on lymphadenopathy [9]. Furthermore, there may be another cause in that patients with HNL might seek medical assistance earlier because of LN tenderness [9].

To establish a simple method for the differential diagnosis of HNL, we used a limited number of clinical findings by selecting factors with an AUC >0.95. However, significantly different factors between HNL and ML that were nonetheless excluded could provide us with insights such as the following to further assess the biological features of these two diseases. Regarding LN tenderness, this study showed that more HNL patients complained of LN tenderness compared with ML patients, which was consistent with previous reports that lymphadenopathy in ML is generally painless [23], whereas painful lymphadenopathy is a characteristic finding of HNL [3]. Although LN tenderness is a subjective finding, and therefore not suitable for differential diagnosis, it may be of interest in identifying which feature of HNL makes lymphadenopathy painful. Both HNL [24] and ML [14] were reported to show high levels of AST. However, the mechanisms underlying this increase in AST have not been identified in HNL or ML, and further study is required. In the present study, higher LDH was observed in HNL compared with ML. One of the possible reasons for this may be the release into the blood of intracellular LDH that results from cell necrosis in the lymph nodes [25]. Regarding ferritin, WBCs, neutrophils, lymphocytes, RBCs, and Plts, we speculate that hemophagocytic syndrome in HNL patients might be the cause of the differences in these factors between HNL and ML because HNL is reported to occasionally become severe and progress to hemophagocytic syndrome [14,26], and the characteristics of hemophagocytic syndrome are an increase in ferritin and a decrease in various types of blood cells [27]. Although the HNL patients enrolled in this study did not undergo bone marrow examination and therefore a definitive diagnosis was not made, they might experience progression to hemophagocytic syndrome. Finally, to the best of our knowledge, there has been no report that compares Na between HNL and ML.

The standardized uptake value (SUV), which reflects nuclide accumulation and is measured by FDG-PET/CT, is reported to be useful in discriminating HNL from indolent ML, where the SUV is higher in HNL than in indolent ML [28]. However, our pilot study performed in 7 HNL patients and 13 ML patients revealed that there was no significant difference between them (median (IQR): 10.30 (8.55, 11.10) in HNL vs. 10.20 (5.80, 12.70) in ML, *p* = 0.9385). The reason why no significant difference was observed between HNL and ML in the current study seems to be that no indolent ML was included and all 13 cases of ML were aggressive, in which the SUVs were comparably high. Even though FDG-PET/CT is a useful tool for identifying the localization of primary tumors and metastatic regions in ML patients, as well as measuring the size of foci, it does not appear to be suitable for distinguishing ML from HNL in children, probably because almost all childhood ML cases are not indolent but rather are of aggressive types [29].

There are several limitations to this study. Because this is a retrospective study, there may be some bias in the population. For example, although 34% of ML cases are at Stage 4 [30], only two Stage 4 cases were included in this cohort (15.4%). Another example was the difference in LDH in ML patients in this study compared with a previous report. Although 45% of ML patients exhibited LDH levels higher than the institutional upper limit of normal (ULN) [31], the percentage of ML patients whose LDH was in excess of the ULN was only 15.4% (2/13 cases) in the present study. Furthermore, the verification of this scoring model using a higher number of pediatric cases with fever and lymphadenopathy is needed before considering its actual implementation. To improve the reliability of this method, all pathologies that are considered in the differential diagnosis of HNL, such as Kawasaki disease, juvenile idiopathic arthritis, or suppurative cervical lymphadenitis, should be considered. Finally, a large, multicenter prospective analysis is also required.

In summary, this study indicates the possibility of a new, noninvasive differential diagnostic method that distinguishes HNL from ML by scoring a combination of three factors, namely body temperature ≥37.8°C, serum BMG level ≥1.8 mg/L, and LN diameter size ≤3.2 cm.

## Figures and Tables

**Table 1:** Comparisons of clinical findings between HNL patients and ML patients.

	ML (*n* = 13)	HNL (*n* = 15)	*p*-Value
**Body temperature (°C), median (IQR)**	**36.90 (36.60, 37.20)**	**39.00 (38.05, 39.15)**	**<0.001**
**Duration of fever (days), median (IQR)**	**0.00 (0.00, 0.00)**	**14.00 (9.00, 19.00)**	**<0.001**
**LN in size (cm), median (IQR)**	**4.00 (3.45, 5.95)**	**1.90 (1.55, 2.00)**	**<0.001**
**Presence of LN tenderness, No (%)**	**2 (15.4)**	**15 (100.0)**	**<0.001**
Skin rash	0 (0)	2 (13.3)	0.4841

Factors with a significant difference (*p*-value < 0.05) are indicated in bold. HNL, histiocytic necrotizing lymphadenitis; ML, malignant lymphoma; IQR, interquartile range; LN, lymph node.

**Table 2:** Comparisons of blood examination parameters between HNL patients and ML patients.

	ML (*n* = 13)	HNL (*n* = 15)	*p*-Value
**WBC (/μL)**	**7400.00 (5800.00, 9700.00)**	**2600.00 (2200.00, 3750.00)**	**<0.001**
**Neutrophil (/μL)**	**4030.00 (2020.00, 4320.00)**	**1711.00 (1182.50, 2087.50)**	**<0.005**
Monocyte (/μL)	248.00 (140.00, 481.00)	152.00 (94.75, 395.00)	0.4327
**Lymphocyte (/μL)**	**3040.00 (1450.00, 3626.00)**	**1189.00 (603.50, 1507.50)**	**<0.01**
Atypical lymphocyte (/μL)	0.00 (0.00, 0.00)	0.00 (0.00, 17.25)	0.5963
**RBC (M/μL** **)**	**5.02 (4.76, 5.15)**	**4.64 (4.38, 4.80)**	**0.0295**
Hb (g/dL)	13.70 (12.60, 14.00)	12.60 (11.65, 13.50)	0.1658
HCT (%)	39.40 (36.80, 41.50)	35.70 (34.50, 39.05)	0.0779
MCV (fL)	78.50 (75.50, 83.60)	80.00 (77.45, 82.85)	0.8301
RDW (fL)	38.10 (36.20, 38.20)	36.70 (35.30, 37.50)	0.1656
**Plt (10^9^/L)**	**2840.00 (2480.00, 3480.00)**	**1710.00 (1525.00, 1940.00)**	**<0.001**
**AST (U/L)**	**22.00 (18.00, 27.00)**	**43.00 (29.00, 55.50)**	**<0.01**
ALT (U/L)	17.00 (12.00, 18.00)	22.00 (14.50, 30.50)	0.1801
TG (mg/dL)	73.00 (56.00, 91.00)	90.00 (61.00, 104.50)	0.2786
**LDH (U/L)**	**260.00 (210.00, 319.00)**	**449.00 (307.00, 617.50)**	**0.01116**
**Na (mEq/L)**	**140.00 (140.00, 140.00)**	**138.00 (135.50, 140.00)**	**<0.01**
CRP (mg/dL)	0.07 (0.03, 0.28)	0.34 (0.16, 0.87)	0.06475
**BMG (mg/L)**	**1.60 (1.30, 1.70)**	**2.50 (2.25, 2.80)**	**<0.001**
**Ferritin (ng/mL)**	**35.00 (28.00, 192.00)**	**282.00 (102.00, 382.00)**	**<0.001**
sIL-2R (U/mL)	684.00 (550.00, 1358.00)	798.00 (654.50, 1033.50)	0.751

Factors with a significant difference (*p*-value < 0.05) are indicated in bold. Data are presented as the median (interquartile range). HNL, histiocytic necrotizing lymphadenitis; ML, malignant lymphoma; WBC, white blood cell count; RBC, red blood cell count, Hb, hemoglobin; HCT, hematocrit; MCV, mean corpuscular volume; RDW, red blood cell distribution width; Plt, blood platelet count; AST, aspartate transaminase; ALT, alanine aminotransferase; TG, triacylglycerol; LDH, lactic dehydrogenase; CRP, C-reactive protein; BMG, β_2_-microglobulin; sIL-2R, soluble IL-2 receptor.

**Table 3:** Cut-off and AUC values of 13 candidate factors for the establishment of a new scoring model for HNL and ML diagnosis.

**HNL > ML**	**Cut-Off**	**AUC**
**Body temperature (°C)**	**37.8**	**0.9641**
Tenderness of LN (%)	50	0.9231
AST (U/L)	23.3	0.7897
LDH (U/L)	292	0.7795
Ferritin (ng/mL)	53	0.8538
**BMG (mg/L)**	**1.8**	**0.9538**
**HNL < ML**	**Cut-Off**	**AUC**
**LN in size (cm)**	**3.2**	**0.9641**
WBC (/μL)	5600	0.9436
Neutrophil (/μL)	3554	0.8051
Lymphocyte (/μL)	2632.5	0.7897
RBC	4.965	0.741
Plt (10^9^/L)	2445	0.8564
Na (mEq/L)	138.5	0.7923

ROC curves revealed three high-precision factors for which the AUC value exceeded 0.95, and the cut-off value for each was then calculated. These factors were body temperature, serum BMG level, and LN size, and the cut-off values to distinguish HNL from ML were ≥37.8°C, ≥1.8 mg/L, and ≤3.2 cm, respectively. Factors demonstrating an AUC of more than 0.95 are indicated in bold. AUC, area under the ROC curve; HNL, histiocytic necrotizing lymphadenitis; ML, malignant lymphoma; LN, lymph node; AST, aspartate transaminase; LDH, lactic dehydrogenase; BMG, β_2_-microglobulin; WBC, white blood cell count; RBC, red blood cell count; Plt, blood platelet count.

**Table 4:** Application of the new scoring model.

	<2	2≤	Total
**HNL**	0	15	15
**ML**	13	0	13
**Total**	13	15	28

The total score of each case ranged from 0 to 3 points because patients were given one point for each factor that satisfied the following conditions: body temperature ≥37.8 °C, serum BMG level ≥1.8 mg/L, and LN diameter ≤3.2 cm. This new scoring model could distinguish HNL from ML with 100% sensitivity, 100% specificity, 100% positive predictive value, and 100% negative predictive value. HNL, histiocytic necrotizing lymphadenitis; ML, malignant lymphoma.

## Data Availability

The datasets used and analyzed during this study can be found in the Kansai Medical University Research Data Storage and are available from the corresponding author upon reasonable request. The data are not publicly available because of privacy restrictions.
